# Safety and Tolerability, Dose-Escalating, Double-Blind Trial of Oral Mannitol in Parkinson's Disease

**DOI:** 10.3389/fneur.2021.716126

**Published:** 2022-01-03

**Authors:** Eduard Linetsky, Suaad Abd Elhadi, Max Bauer, Akiva Gallant, Montaser Namnah, Sagit Weiss, Daniel Segal, Ronit Sharon, David Arkadir

**Affiliations:** ^1^Department of Neurology, Faculty of Medicine, Hadassah Medical Organization, Hebrew University, Jerusalem, Israel; ^2^Department of Biochemistry and Molecular Biology, Faculty of Medicine, Institute for Medical Research Israel-Canada (IMRIC), The Hebrew University of Jerusalem, Jerusalem, Israel; ^3^Crowdacure LTD, London, United Kingdom; ^4^Shmunis School of Biomedicine and Cancer Research, Tel Aviv University, Tel Aviv, Israel

**Keywords:** Parkinson's disease, safety, tolerability, clinical trial, mannitol

## Abstract

Mannitol, a natural alcoholic-sugar, was recently suggested as a potential disease-modifying agent in Parkinson's disease. In animal models of the disease, mannitol interferes with the formation of α-synuclein fibrils, inhibits the formation of α-synuclein oligomers and leads to phenotypic recovery of impaired motor functions. Parkinson's patients who consume mannitol report improvements of both motor and non-motor symptoms. Safety of long-term use of oral mannitol, tolerable dose and possible benefit, however, were never clinically studied. We studied the safety of oral mannitol in Parkinson's disease and assessed the maximal tolerable oral dose by conducting a phase IIa, randomized, double-blind, placebo-controlled, single-center, dose-escalating study (ClinicalTrials.gov Identifier: NCT03823638). The study lasted 36 weeks and included four dose escalations of oral mannitol or dextrose to a maximal dose of 18 g per day. The primary outcome was the safety of oral mannitol, as assessed by the number of adverse events and abnormal laboratory results. Clinical and biochemical efficacy measures were collected but were not statistically-powered. Fourteen patients receiving mannitol completed the trial (in addition to eight patients on placebo). Mannitol-related severe adverse events were not observed. Gastrointestinal symptoms limited dose escalation in 6/14 participants on mannitol. None of the clinical or biochemical efficacy secondary outcome measures significantly differed between groups. We concluded that long-term use of 18 g per day of oral mannitol is safe in Parkinson's disease patients but only two third of patients tolerate this maximal dose. These findings should be considered in the design of future efficacy trials.

## Introduction

Current therapies for Parkinson's disease (PD) aim to alleviate symptoms. In the search for disease-modifying therapies in PD, numerous generic and non-patentable agents have been suggested (1, 2). However, only a relatively small number of these agents were tested in large scale double-blind trials (3, 4). Mannitol, a 6-carbon polyol that was recently suggested as a potential disease-modifying agent (5) was never clinically studied in PD patients.

*In vitro*, it has been shown that mannitol interferes with the formation of α-synuclein fibrils (5). At high doses mannitol also inhibits the formation of α-synuclein oligomers, a form that may be even more toxic to neurons. More impressively, exposure of a fruit fly model of PD (transgenic α-synuclein A53T) to a medium containing mannitol led to a full recovery of impaired motor functions. These observations, which were received with great enthusiasm by PD patients, led many individuals affected by the disease to begin consuming daily oral mannitol. Self-reported outcomes included improved sense of smell, reduction in the dose of PD medications and general improvement in well-being. Lack of data regarding the safety of long-term use of high-dose oral mannitol motivated us to conduct a double-blind study of the safety and tolerability of this agent. We also searched for hints of disease-modification, although our study was not statistically powered for this.

## Materials and Methods

### Study Design

This is a randomized, double-blind, placebo-controlled, single-center, dose-escalating study lasting 36 weeks (ClinicalTrials.gov Identifier: NCT03823638). The work has been carried out in accordance with The Code of Ethics of the World Medical Association (Declaration of Helsinki) for experiments involving humans. The study was approved by the institutional IRB committee (0346-17-HMO). All participants signed an informed consent before any trial-related procedure was performed.

Patients were recruited at the clinic for Parkinson's Disease and Movement Disorders at Hadassah Medical Center (Jerusalem, Israel) between November 2018 and March 2020. The clinical study included five visits at the clinic. In the first visit, patients were screened (see inclusion and exclusion criteria below) and were then randomly assigned to receive either oral mannitol (2.5 g twice daily, manufacturer Roquette, France) or oral placebo (2.5 g twice daily of dextrose, manufacturer Roquette, France). To achieve allocation concealment participants were assigned to either the mannitol or the placebo arms by a blinded team member who used a randomization table. In visits 2–4 (intervals of 6, 6, and 12 weeks respectively) the doses of the investigational product were gradually increased (4, 6, and 9 g twice daily, respectively). In cases of clinically significant side effects, the dose was adjusted to the highest previous tolerable dose. The trial was completed 12 weeks later (visit five).

During visits 1–4 participants received a container with the investigational product and a measuring tube marked for the relevant volume of powder. Participants were instructed to dissolve, the measured volume powder in water, twice daily, and consume it. Adherence was documented twice daily by a self-reported table. During these visits, the anti-parkinsonian treatment could be changed by the investigator if needed.

### Subjects

Participants were 40–79 years of age who were diagnosed with PD after the age of 40 years, based on the United Kingdom Brain Bank criteria. Patients were cognitively preserved (Montreal Cognitive Assessment score ≥25/30) and were on a stable regimen of anti-Parkinsonian medications for at least 4 weeks. Exclusion criteria included symptomatic therapy taken more than four times a day, advanced PD therapies (subcutaneous apomorphine, deep brain stimulation or levodopa continuous intrajejunal infusion), history of psychosis or the use of dopamine receptor blocking agents on the year proceeding the trial, suspected Parkinsonian syndrome other than PD, pregnancy, diabetes mellitus, congestive heart failure or symptomatic orthostatic hypotension.

### Outcomes

The primary outcome of the study was the safety of oral mannitol in PD as assessed by the number of mannitol-related adverse events, clinically significant changes in vital signs and clinically significant abnormalities in laboratory results.

Secondary outcome measures between visits one and five (week 36) included: 1) Time-interval for starting symptomatic therapy in patients not receiving symptomatic therapy at baseline; 2) Change in daily levodopa-equivalent dose (LED) units; 3) Change of Brief Smell Identification Test (B-SIT) score, 4) Change in Constipation Assessment Scale (CAS); 5) Change in Montreal Cognitive Assessment (MoCA) test score; 6) Change in Non-Motor Symptoms of Scale for Parkinson's disease (NMSS); and 7) Change in the ratio of total-to-proteinase K-resistant α-synuclein in red blood cells (RBC) measured by enzyme-linked immunosorbent assay (ELISA). Motor scores were documented during the visits, but were not part of the outcome measures, as patients were not asked to avoid medication on the night before clinic visits.

### Sample Size and Statistical Analysis

We targeted a sample size of 25 per group, with assumed 7% dropouts unrelated to agent tolerability. This sample size provided 80% power to detect a difference of 30% in the proportion of subjects tolerating mannitol relative to placebo (using a 1-sided *t*-test with alpha <0.05), assuming the placebo group will have more than 90% tolerability.

Efficacy measures, calculated only for those patients who completed the treatment originally allocated, were not powered to detect statistically significant differences, but rather to be used for designing future efficacy studies with the maximal tolerable dose of mannitol that will be defined in this study. The targeted sample size (including the 7% dropouts), however, would have enabled us to detect inter-group differences of 20% in the mean of continuous variables (secondary objectives) with 80% power in 2-sided *t*-test with alpha <0.05 in case the intra-group standard deviation would be 24%. Differences between groups were assessed using two-sided Wilcoxon rank sum test.

The COVID-19 pandemic in 2020 dramatically slowed recruitment rate in the 3rd year of the clinical trial and led to the decision of earlier trial termination.

### Biochemical Measures

Blood samples were collected in EDTA tubes and processed within 2–3 h from collection as previously described (6). The levels of total α-synuclein and phosphorylated α-synuclein (pSer129) in lysed blood cells were determined (6). To measure the total and pSer129 α-synuclein levels in the membrane, pellets were obtained following osmotic cell lyses of blood cells. Membrane pellets were washed three times in DDW to remove cells and soluble proteins and membrane proteins were extracted in STET buffer (10 mM Tris-HCl, 1 mM EDTA, 100 mM NaCl) containing 1% Triton X-100 (v/v) with proteinase inhibitors (30 min on ice). Samples were then spun at 14,000 RPM for 10 min and the supernatant was collected for analyses. Samples were diluted 1:100 with 1% BSA (fatty acid free) in PBS and were applied to a microtiter plate at 15 and 30 μl.

To measure phosphatidylinositol 4,5-bisphosphate (PI4,5P_2_) levels, membrane pellets were washed three times with DDW and liquids were completely removed. Total lipids were extracted in cold chloroform: methanol (1:1) using the Folch method (7) with the addition of 30 μM AlCl_3._ Samples were vortexed for 1 min and centrifuged at 9,000 RPM for 5 min. The pellet fraction obtained from the resultant extraction (1st extraction) was dissolved in chloroform: methanol 2:1 with AlCl_3_ and 0.25% HCl, vortexed for 5 min and centrifuged at 13,000 RPM for 5 min. The lower phase of this extraction (2nd extraction) was used for analysis of PI4,5P_2_ levels. In addition, samples of both 1st and 2nd extractions were diluted in methanol at 1:30 or 1:3, respectively, and applied on a microtiter plate at 10–40 μl per well. A standard curve consisting of purified PI4,5P_2_ (Echelon) was applied in parallel at 0–60 ng/well. Solvents were dried under N_2_ and PI4,5P_2_ levels were detected using anti PI4,5P_2_ antibodies (Echelon).

## Results

### Characteristics of the Subjects

**Figure 1:**
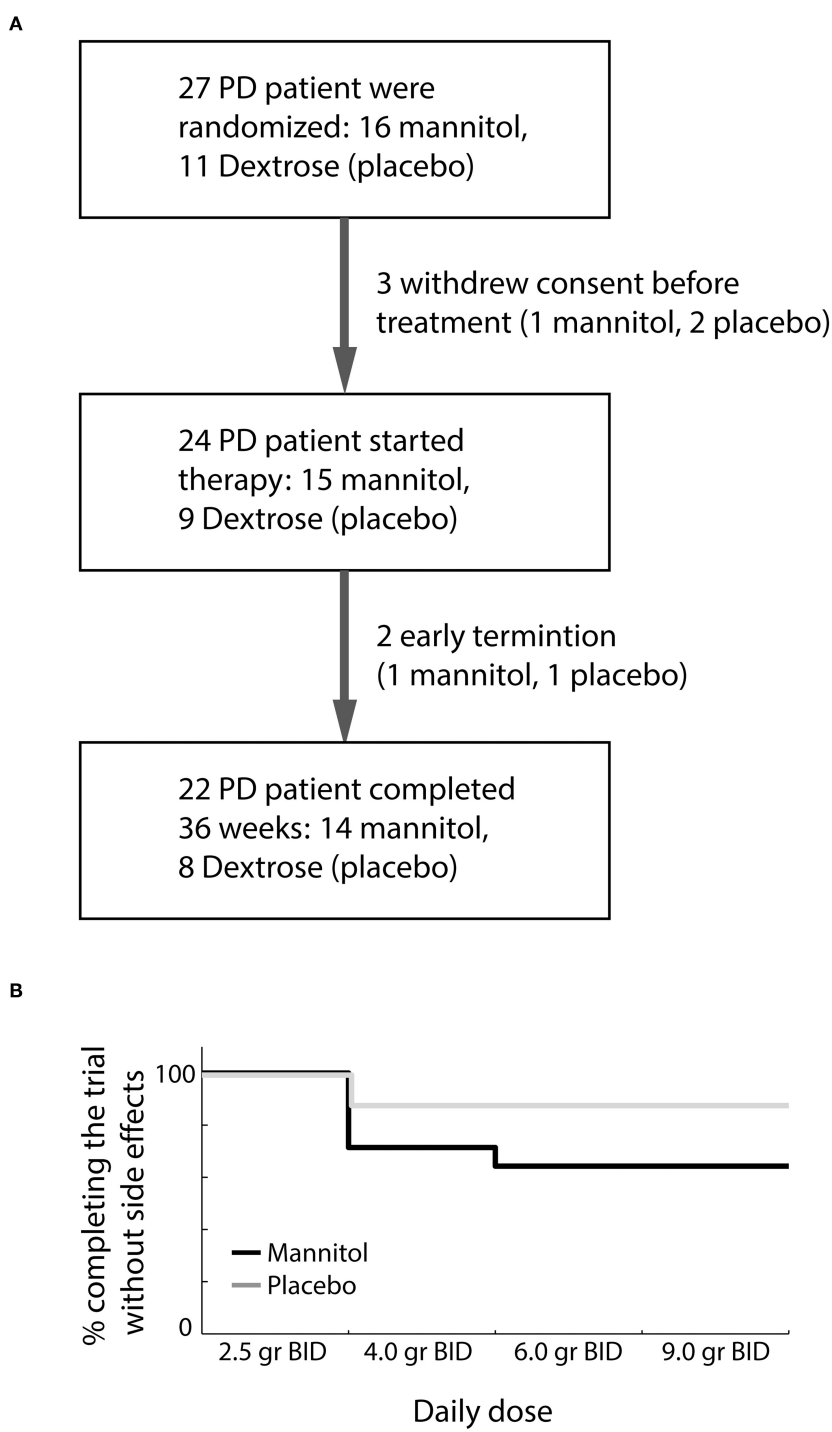
Trial flow diagram and clinical outcome. **(A)** First trial visit included both screening for eligibility and randomization. **(B)** Dosage tolerability among participants who completed the trial in the mannitol (black) and placebo (dextrose, gray) groups.

A total of 27 participants signed the IRB-approved informed consent and were randomized to blindly receive oral mannitol or placebo (Figure 1A). Three participants withdrew their consent before initiation of therapy. Baseline demographic and clinical characteristics of randomized subjects were similar between the two groups (Table 1). The adherence of participants to the investigation product was high in both groups (mannitol: 89–100%, median 99%; placebo: 91–100%, median 100%).

**Table 1:** Baseline demographic and clinical characteristics of randomized subjects.

	**Placebo (*n* = 11)**	**Mannitol (*n* = 16)**	***P* Value**
Age (Years)	68.5 (48–76.5)	66.7 (52.5–75.2)	0.47
Female / Male	5/6	6/10	0.71
Years from diagnosis	2 (0–8)	2 (0–15)	0.96
Body mass index (BMI)	26.6 (19.3–32.4)	25.6 (18.6–32.5)	0.71
Individuals on levodopa	8/11	10/16	1.00
Daily LED	360 (100–500)	300 (0–625)	0.37
B-SIT	6 (2–10)	6 (2–12)	0.55
CAS	1 (0–5)	0.5 (0–7)	0.61
MoCA	29 (26–30)	28 (26–30)	0.26
NMSS	10 (3–31)	9.5 (0–98)	0.57
ADL	90 (80–100)	90 (80–90)	0.61
H&Y	2 (1–2.5)	2 (1–3)	0.35
UPDRS I	0 (0–3)	1 (0–3)	0.49
UPDRS II	6 (3–13)	5.5 (1–16)	0.84
UPDRS III	17 (7–35)	19 (11–30)	0.30

*LED, Daily Levodopa Equivalent Dose; B-SIT, Brief Smell Identification Test; CAS, Constipation Assessment Scale; MoCA, Montreal Cognitive Assessment scale, NMSS, Non-Motor Symptoms Scale; ADL, Schwab & England Activities of Daily Living scale; H&Y, Hoehn and Yahr scale; UPDRS, Unified Parkinson's Disease Rating Scale*.

### Primary Clinical Outcome Measures (Safety and Tolerability)

Twenty-four participants initiated oral therapy (15 mannitol, 9 placebo). One participant from each of the groups terminated the study earlier than planned. Early termination of a participant receiving mannitol was due to *in situ* breast carcinoma discovered 3 weeks after the first visit. This severe adverse event was considered unrelated to mannitol treatment. The participant in the placebo group dropped out of the trial within a week due to nausea. This side effect was considered as related to the investigated product by the investigator.

No mannitol-related severe adverse events occurred during the study. A single severe adverse event (recurrence of prostate carcinoma), considered unrelated to the investigated product, was documented on the last visit in one patient receiving placebo. Clinically significant gastrointestinal symptoms (diarrhea, nausea, abdominal discomfort) were reported in 6/14 participants in the mannitol group who completed the study. In 5/14 participants these symptoms required dose reduction (Figure 1B). Only a single participant in the placebo group required dose reduction due to abdominal discomfort (Fisher exact *P* = 0.19). Overall, 64% of participants receiving mannitol tolerated well the target dose of 18 g of mannitol per day divided into two doses.

Blood tests, taken during each of the visits before dose increment, did not reveal electrolyte abnormalities, increased liver enzymes, impaired renal function, elevated non-fasting glucose level or evidence of systemic inflammation (leukocytosis or increased CRP). Blood counts and biochemistry parameters did not change during the trials and no adverse events were documented by the laboratory tests (Table 2).

**Table 2:** Values of whole-blood laboratory parameters at the final visit of participants receiving placebo (dextrose) or mannitol.

	**Placebo, last visit (*n* = 11)**	**Mannitol, last visit (*n* = 14)**	***P* Value**
Creatinine	49–107 (75.5) μmol/L	45–110 (81.5) μmol/L	0.45
Urea	2.8–7.6 (3.8) mmol/L	2.0–7.1 (3.8) mmol/L	0.89
Sodium	135–141 (138) mmol/L	131–142 (135) mEq/L	0.30
C reactive protein	>0.05–1.2 (0.05) mg/L	>0.05–1.0 (0.07) mg/L	0.71
Hemoglobin	10.6–15.6 (13.8) g/dL	12.5–15.6 (14.0) g/dL	0.56
Non fasting glucose	4.3–5.9 (5.1) mmol/L	4.7–6.2 (5.2) mmol/L	0.61

*Minimum - maximum and (median) values are presented*.

### Secondary Clinical Outcome Measures (Efficacy)

The study was not designed to demonstrate statistically significant differences in efficacy measures. None of the clinical measures were significantly different between the mannitol and placebo groups (Table 3). Sub-analysis, comparing the placebo group with participants who completed the trial on the maximal target dose of mannitol (18 g, *n* = 9), also did not reveal significant differences. Only a single participant (mannitol group), not on levodopa at screening, started levodopa therapy during the trial.

**Table 3:** Change of clinical outcome measures of efficacy between baseline and 36 weeks of continuous therapy.

	**Placebo (*n* = 8)**	**Mannitol (*n* = 14)**	***P* Value**	**Full dose Mannitol (*n* = 9)**	***P* Value**
**Secondary clinical outcome measures**					
LED	0 (−75–0)	0 (−225–50)	0.66	0 (−75–0)	1.00
B-SIT	−1.5 (−4–1)	−1 (−5–3)	0.20	−1 (−3–3)	0.35
MoCA	0 (−2–1)	−1 (−3–5)	0.81	−1 (−1–5)	0.66
NMSS	−4 (−12–13)	0 (−12–20)	0.37	0 (−12–20)	0.52
CAS	0 (−2–1)	0 (−3–3)	0.94	0 (−3–3)	0.72
**Other clinical outcome measures**					
ADL	0 (0–10)	0 (0–10)	0.65	0 (0–10)	0.67
UPDRS I	0 (−1–2)	0 (−1–6)	0.64	0 (−1–6)	0.89
UPDRS II	0.5 (−3–1)	0 (−1–5)	0.70	0 (−1–3)	0.77
	**Placebo (*****n*** **=** **2)**	**Mannitol (*****n*** **=** **6)**	***P*** **Value**	**Full dose Mannitol (*****n*** **=** **5)**	***P*** **Value**
**Other clinical outcome measures in participants without levodopa**					
H&Y	0.3 (0–0.5)	0 (0–0.5)	0.99	0 (0–0.5)	1.00
UPDRS III	0 (−6–6)	−1.5 (−18–5)	0.64	−1 (−18–5)	0.57
UPDRS I-III	−1 (−5–3)	−2 (−16–13)	1.00	−1 (−16–13)	1.00

*LED, Daily Levodopa Equivalent Dose; B-SIT, Brief Smell Identification Test; CAS, Constipation Assessment Scale; MoCA, Montreal Cognitive Assessment scale; NMSS, Non-Motor Symptoms Scale; ADL, Schwab & England Activities of Daily Living scale; H&Y, Hoehn and Yahr scale; UPDRS, Unified Parkinson's Disease Rating Scale*.

### Biomarker Measures

Blood samples taken with each increment of mannitol or placebo dose were used to measure biochemical variables that included total and pSer129 α-synuclein levels, in both lysed blood cells and in membrane pellets of lysed blood cells (6), and PI4,5P_2_ levels that based on recent findings are increased in PD models (8, 9). No observable or statistically significant differences were demonstrated between the groups and no intra-group longitudinal changes were observed during the trial.

### Retrospective Power Calculation for Future Trials

PD patients who buy and consume oral mannitol (not as part of controlled clinical studies) frequently report improvement of their sense of smell. In our small study no such effect was observed (in the mannitol group the median change in B-SIT score was −1, a mean change of −0.54 ± 2.22). Based on these results, a future study aiming to demonstrate an improvement of at least 1 point on the B-SIT test, with a power of 80% and a significance level of 5% in a one-sided paired *t*-test, would require a sample size of at least 32 subjects on mannitol.

## Discussion

Oral mannitol is absorbed through non-mediated diffusion by small channels in enterocyte brush border membrane. It is estimated that the oral bioavailability of mannitol ranges from as low as 20% (10) to as high as 60% (11). Absorption may be lower in patients with Parkinson's disease (12). While the permeability of the blood-brain-barrier (BBB) to mannitol is relatively low, data in animal models indicates that mannitol penetrates the brain (13).

The mechanism by which mannitol reduces α-synuclein accumulation in PD models is still unknown. It has been shown that mannitol increases the expression of 70 kilodalton heat shock proteins (HSP-70) and therefore, potentially facilitates correct folding of α-synuclein and removal of misfolded forms (5). Another possible mechanism comprises the property of mannitol as a scavenger of free radicals (14). Our view is that the mechanism by which a single, high-dose intravenous application of mannitol reduces intracranial pressure, namely osmolar pressure, does not play a role in the reduction of α-synuclein accumulation.

Our study was not powered to demonstrate efficacy. Following 36 weeks of exposure to mannitol we did not observe a clear reduction in Parkinson's symptoms that were previously reported by patients taking mannitol (not in a clinical study). It is possible that a longer exposure would enable clinically to demonstrate disease modification that was pathologically observed in a mouse of PD after only 4 weeks of exposure (5). Long study would also enable to demonstrate clinical deterioration in the placebo group.

This study established the safety of long-term use of oral mannitol in doses up to 18 grams per day. Larger clinical trials that would target efficacy would have to take into account that only two thirds of participants tolerated this dose. The levels of mannitol in the cerebrospinal fluid following exposure to such oral doses should be measured and compared to the levels shown effective in PD animal models (5) before a larger efficacy study will be considered.

## Data Availability Statement

The raw data supporting the conclusions of this article will be made available by the authors, without undue reservation.

## Ethics Statement

The studies involving human participants were reviewed and approved by Hadassah Medical Organization approval 0346-17-HMO. The patients/participants provided their written informed consent to participate in this study.

## Author Contributions

EL, MB, and MN: substantial contributions to the acquisition of data. SA: substantial contributions to the acquisition and analysis of data. AG: substantial contributions to the analysis of data and draft the manuscript. SW: substantial contributions to interpretation of data and revising the draft. DS: substantial contributions to the conception of this work. RS: substantial contributions to the conception of the work, analysis of data, and drafting the manuscript. DA: substantial contributions to the conception and design of the work and the acquisition, analysis and interpretation of data, and drafting the work. All authors agree to be accountable for all aspects of the work in ensuring that questions related to the accuracy or integrity of any part of the work are appropriately investigated and resolved, revised, and approved the final manuscript.

## Funding

This work was supported by the Israeli Ministry of Science and Technology grant for non-patentable drugs (grant 3-14729).

## Conflict of Interest

SW is the director of Crowdacure LTD. The remaining authors declare that the research was conducted in the absence of any commercial or financial relationships that could be construed as a potential conflict of interest.

## Publisher's Note

All claims expressed in this article are solely those of the authors and do not necessarily represent those of their affiliated organizations, or those of the publisher, the editors and the reviewers. Any product that may be evaluated in this article, or claim that may be made by its manufacturer, is not guaranteed or endorsed by the publisher.

## References

[1] Du Y Ma Z Lin S Dodel RC Gao F Bales KR. Minocycline prevents nigrostriatal dopaminergic neurodegeneration in the MPTP model of Parkinson's disease. Proc Natl Acad Sci U S A. (2001) 98:14669–74. 10.1073/pnas.251341998 11724929 PMC64739

[2] Cai R Zhang Y Simmering JE Schultz JL Li Y Fernandez-Carasa I. Enhancing glycolysis attenuates Parkinson's disease progression in models and clinical databases. J Clin Invest. (2019) 129:4539–49. 10.1172/JCI129987 31524631 PMC6763248

[3] Parkinson Study Group QE3 Investigators Beal MF Oakes D Shoulson I Henchcliffe C Galpern WR Haas R. A randomized clinical trial of high-dosage coenzyme Q10 in early Parkinson disease: no evidence of benefit. JAMA Neurol. (2014) 71:543–52. 10.1001/jamaneurol.2014.131 24664227

[4] Parkinson Study Group SURE-PD. Investigators, Schwarzschild MA, Ascherio A, Beal MF, Cudkowicz ME, Curhan GC, Hare JM, et al. Inosine to increase serum and cerebrospinal fluid urate in Parkinson disease: a randomized clinical trial. JAMA Neurol. (2014) 71:141–50. 10.1001/jamaneurol.2013.5528 24366103 PMC3940333

[5] Shaltiel-Karyo R Frenkel-Pinter M Rockenstein E Patrick C Levy-Sakin M Schiller A. Blood-Brain Barrier (BBB) disrupter is also a potent α-Synuclein aggregation inhibitor: a novel dual mechanism of mannitol for the treatment of Parkinson disease. J Biol Chem. (2013) 288:17579–88. 10.1074/jbc.M112.434787 23637226 PMC3682557

[6] Abd Elhadi S Grigoletto J Poli M Arosio P Arkadir D Sharon R. α-Synuclein in blood cells differentiates Parkinson's disease from healthy controls. Ann Clin Transl Neurol. (2019) 6:2426–36. 10.1002/acn3.50944 31742923 PMC6917335

[7] Folch J Lees M Sloane Stanley GH. A simple method for the isolation and purification of total lipides from animal tissues. J Biol Chem. (1957) 226:497–509. 10.1016/S0021-9258(18)64849-5 13428781

[8] Schechter M Atias M Abd Elhadi S Davidi D Gitler D Sharon R. α-Synuclein facilitates endocytosis by elevating the steady-state levels of phosphatidylinositol 4,5-bisphosphate. J Biol Chem. (2020) 295:18076–90. 10.1074/jbc.RA120.015319 33087443 PMC7939461

[9] Schechter M Grigoletto J Abd-Elhadi S Glickstein H Friedman A Serrano GE. role for α-Synuclein in axon growth and its implications in corticostriatal glutamatergic plasticity in Parkinson's disease. Mol Neurodegeneration. (2020) 15:24. 10.1186/s13024-020-00370-y 32228705 PMC7104492

[10] Nasrallah SM Iber FL. Mannitol absorption and metabolism in man. Am J Med Sci. (1969) 258:80–8. 10.1097/00000441-196908000-00003 4979740

[11] FDA Application number: 22368Orig1s000. Available online at: https://www.accessdata.fda.gov/drugsatfda_docs/nda/2010/022368Orig1s000ClinPharmR.pdf (accessed December 17, 2021).

[12] Davies KN King D Billington D Barrett JA. Intestinal permeability and orocaecal transit time in elderly patients with Parkinson's disease. Postgrad Med J. (1996) 72:164–7. 10.1136/pgmj.72.845.164 8731708 PMC2398397

[13] Amtorp O. Estimation of capillary permeability of inulin, sucrose and mannitol in rat brain cortex. Acta Physiol Scand. (1980) 110:337–42. 10.1111/j.1748-1716.1980.tb06678.x 6786001

[14] André P Villain F. Free radical scavenging properties of mannitol and its role as a constituent of hyaluronic acid fillers: a literature review. Int J Cosmet Sci. (2017) 39:355–60. 10.1111/ics.12386 28027572

